# Supplementary figures and images for: Genomic analysis unveils genome degradation events and gene flux in the emergence and persistence of S. Paratyphi A lineages
Source: PLoS Pathog. 2023 Apr 28;19(4):e1010650. doi: 10.1371/journal.ppat.1010650 (PMC10171690; doi:10.1371/journal.ppat.1010650)

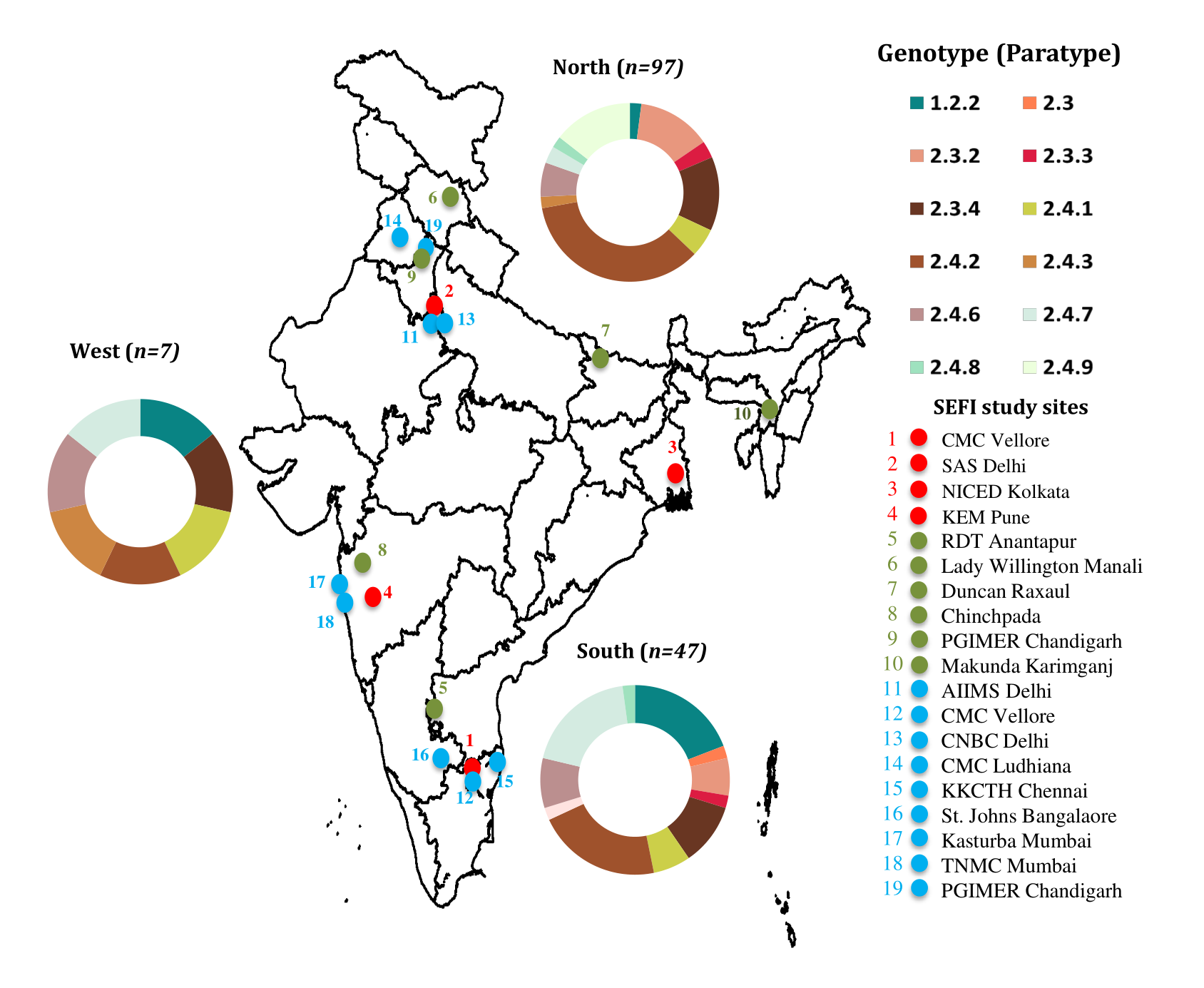

Supplement: S1 Fig — Pie chart colours indicate the propotion of genotypes prevalent in three major geographical locations in India. Study sites are represented as per the settings. Color keys for all the variables are given in the inset legend. Shape file of India containing the boundaries of country, state and UT used with permission from GitHub repository (https://github.com/AnujTiwari/India-State-and-Country-Shapefile-Updated-Jan-2020) under a CC BY license. The base map was created using ArcGIS software by Esri (www.esri.com). ArcGIS and ArcMap are the intellectual property of Esri and are used herein under license. Annotations were added to the base map using Microsoft Powerpoint. (TIF) [file ppat.1010650.s001.tif]

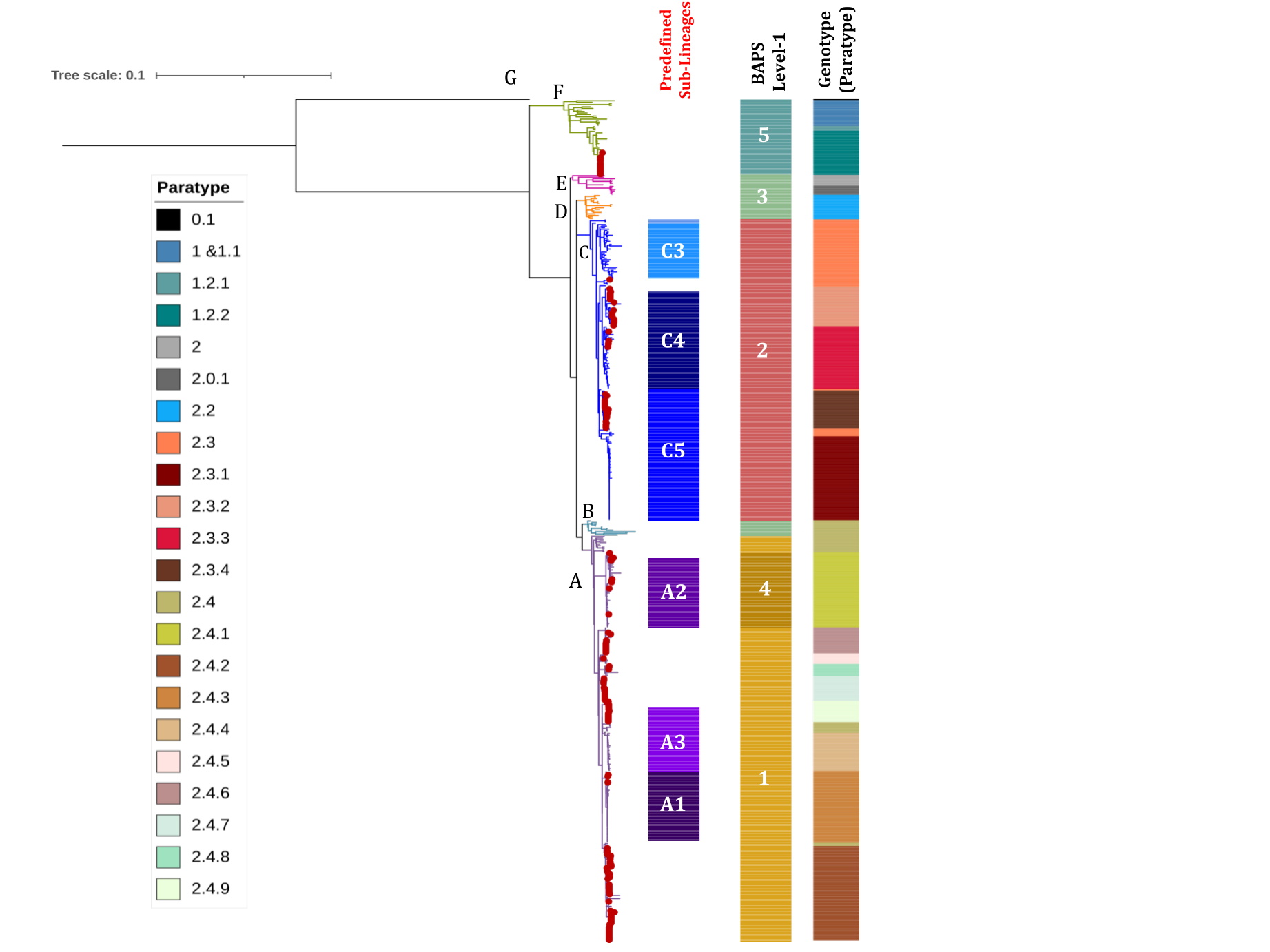

Supplement: S2 Fig — Lineages are represented by various colored branches. Sublineages, BAPS cluster and Paratype scheme are labeled as color strips. (TIF) [file ppat.1010650.s002.tif]

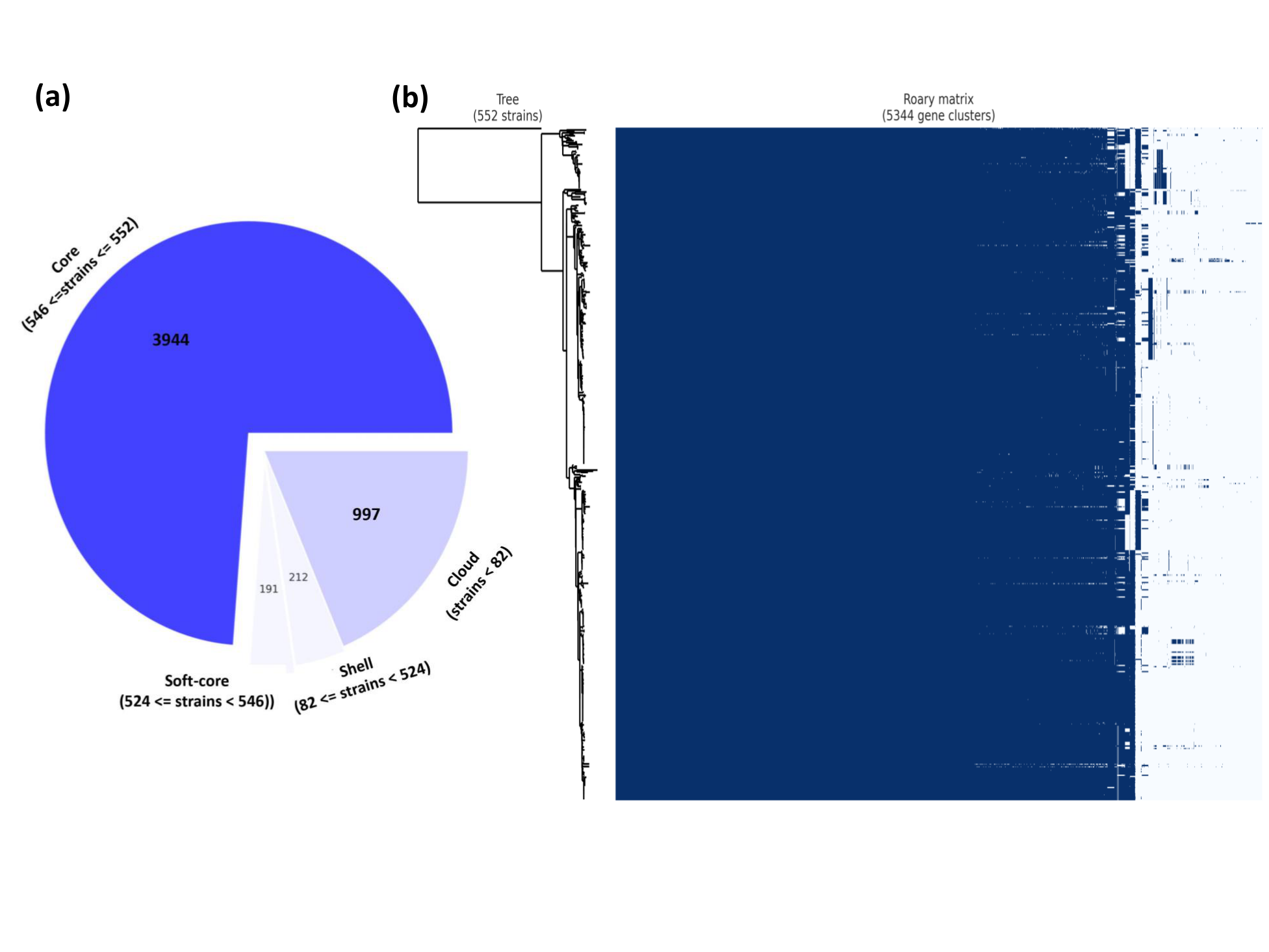

Supplement: S3 Fig — (a) Pie chart indicates the core, soft core, shell and cloud genome composition of S. Paratyphi A genomes (b) Maximum likelihood tree of S. Paratyphi A genomes were compared to a matrix with the presence (blue) and absence (white) of the accessory genes found in the pan-genome. The image was prepared using Phandango (https://jameshadfield.github.io/phandango/#/) (TIF) [file ppat.1010650.s003.tif]

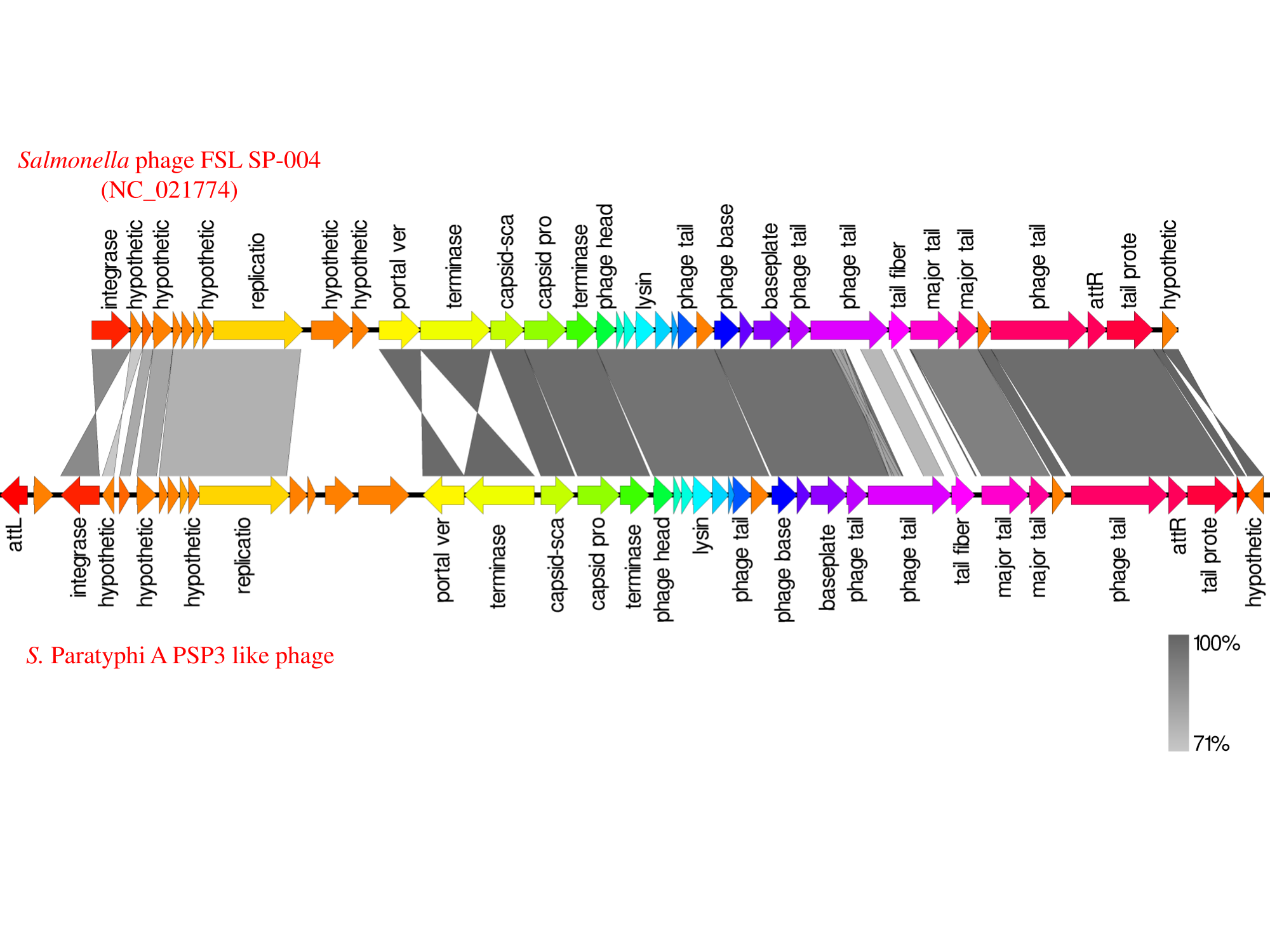

Supplement: S4 Fig — (TIF) [file ppat.1010650.s004.tif]

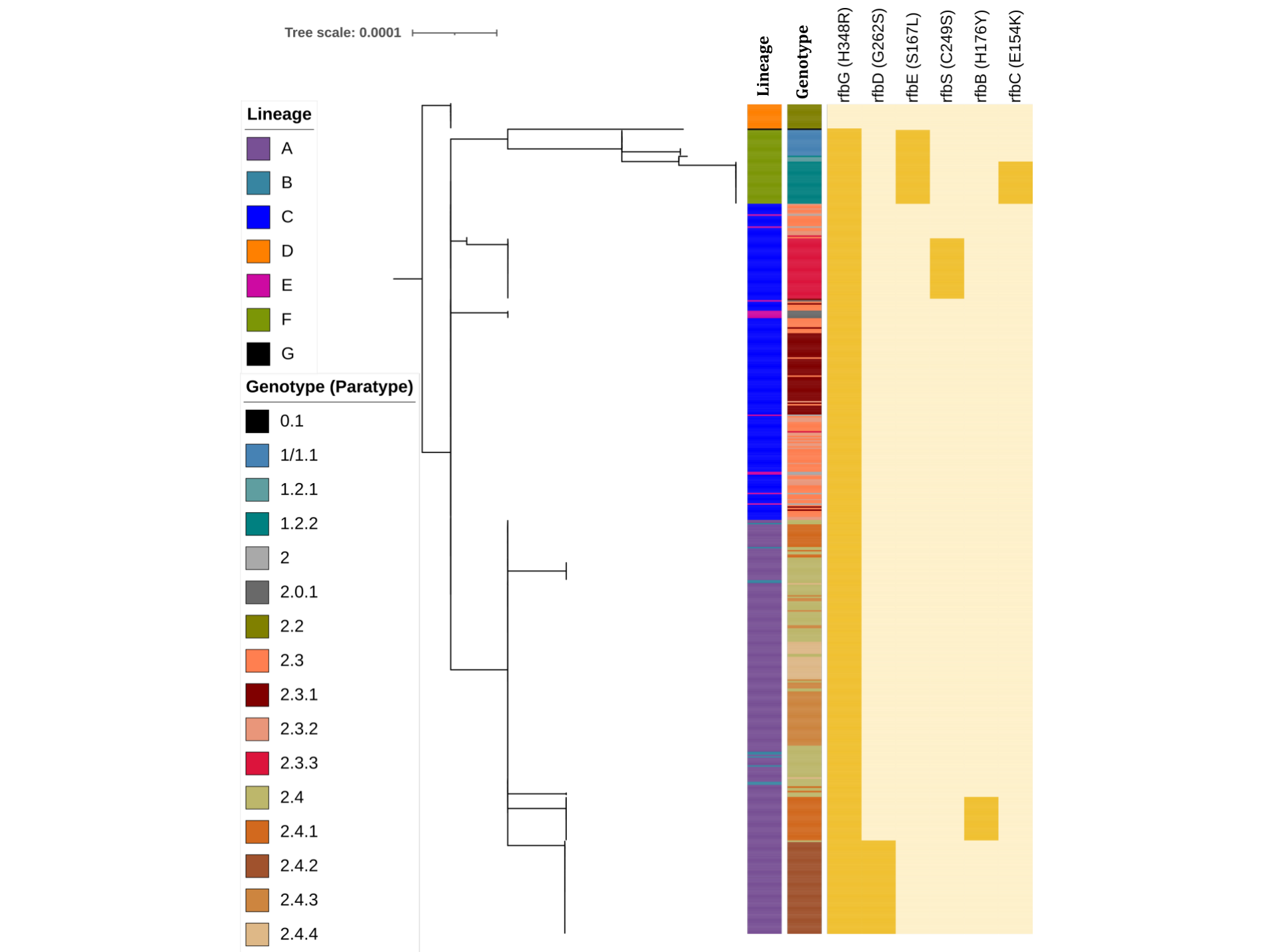

Supplement: S5 Fig — Lineages and genotypes are labelled as colour strips. Amino acid substitutions in the rfb loci are represented by heat maps. (TIF) [file ppat.1010650.s005.tif]
