# Supplementary material for: Genomic analysis unveils genome degradation events and gene flux in the emergence and persistence of S. Paratyphi A lineages
Source: PLoS Pathog. 2023 Apr 28;19(4):e1010650. doi: 10.1371/journal.ppat.1010650 (PMC10171690; doi:10.1371/journal.ppat.1010650)
Supplement: Attachment — Submitted filename: Response to Reviewer comments_V4.docx [file ppat.1010650.s013.docx]

**PPATHOGENS-D-22-01006**

**Reviewer's Responses to Questions**

| **Reviewer comments** | **Response** |
| --- | --- |
| Reviewer #1: |  |
| **Part I – Summary:**  This is an important study looking at the genomic epidemiology and population structure of a relatively neglected pathogen in India. Genome sequencing and appropriate bioinformatic analyses have been used. I'm very happy with the methodology and interpretation of results. The work is very significant in two main areas. Firstly in the insights it gives us into evolution of this pathogen and secondly in its relevance to vaccine/diagnostic testing. Overall the study was well designed and performed. The level of scholarship is what I would expect to see in PLOS Pathogens. | Thank you for your appreciation of our work. We sincerely value the time and effort you have put into the review of our manuscript. |
| **Part II – Major Issues: Key Experiments Required for Acceptance:**  I have no major issues with this paper that need to be addressed. | Thank you. |
| **Part III –** **Minor Issues: Editorial and Data Presentation Modifications:** |  |
| I would like to see a brief description of the sampling framework (geographical distribution, coverage of population) in the introduction or the results. I appreciate that this information is given in methods and can be obtained from other papers describing SEFI but it would help the reader to have this information earlier in the paper.  Line 102 (paragraph). Several means are provided in this paragraph with no indication of range or confidence. Please could the authors provide slightly more information. I appreciate that the details are available in supplementary data but 95% CIs (or ranges) would help indicate if these means a representative or where there is a lot of diversity among sampling sites.  Line 110: The authors write "The antimicrobial susceptibility test demonstrated that 100% of *S*. Paratyphi clinical isolates (n=441) were non-MDR". This would be better stated as "no. *S*. Paratyphi clinical isolates (n=441) were MDR"  Line 114: The authors write "Overall, Indian *S*. Paratyphi A isolates were found to be generally non-susceptible to ciprofloxacin, while they continue to be susceptible to first-line agents." The use of the word "generally" is not acceptable without providing some data. Please provide a proportion and include the denominator. | We thank the suggestion from this reviewer. A brief description of sampling framework including geographical distribution and coverage of population is now been included in Introduction (Line No. 117 - 125) and Methods (Line No. 317 - 323)  We agree that better use of range or confidence would be more accurate and precise for mean or median values. However, in this paragraph the percentage values do not indicate any mean or median. Rather the values represent the proportion of samples among the total number. To give more clarity, we have now included the denominator values as well. (Line No. 132-133)  Thank you for the suggestion. The sentence has now been changed.  Line No. 139 -142  Changes have been made  Line No. 144 |
| Reviewer #2: |  |
| **Part I – Summary:**  Jacob et al describe the population structure, distribution of genotypes within India and sequence polymorphisms associated with potential pseudogenes and AMR in 152 new *S*. Paratyphi A sequences from India in the context of 400 global isolates. The sequence analysis has been carried out to a high standard and the report of the population structure of Paratyphi A from India will significantly increases the number of genome sequences in the public domain for this country and globally. The population structure has been described with a new higher resolution nomenclature called paratype that builds on the population structure described by Zhou et al, 2014. However, beyond a description of the distribution of genotypes in North, South or West India, little new insight into paratyphoid disease in this country is reported and the significance of their distribution is not discussed. Analysis of sequence polymorphisms appears only an incremental advance on that published previously by Zhou et al, 2014 and it not clear from the text if any new potential pseudogenes were identified by this analysis. The title of the manuscript suggests that the role of sequence polymorphisms leading to potential loss-of-function has been investigated but this is not the case. Although their association with certain lineages suggests that they may be important in the success of the genotype, it seems misleading to suggest that their role has been established. Functional analysis of sequence polymorphism was carried out on mis-sense mutations in several rfb genes involved in biosynthesis of long chain LPS, motivated by the fact that these may affect efficacy of potential vaccines targeting LPS antigens. Although computational predictions suggested that most of these would not negatively impact function, experimental confirmation was limited to agglutination with the O2 antigen, despite O12 being the immune dominant antigen for Paratyphi A LPS. | Thank you for your assessment of our work. In this study we intended to present a better resolution to the current genomic framework of *S*. Paratyphi A (proposed by Zhou et al, 2014). Besides the classical seven lineages represented by A-G, we have provided higher resolution to sub-clades or sub-lineages by incorporating Paratype genotyping framework. Additional data including, lineage defining mutations, insertions or deletions can be used for designing primers capable of differentiating lineages.  As pointed out by the reviewer, it would have been interesting to explore the burden of paratyphoid disease in the country. However, the scope and the primary aim of our study was to focus on the genomic epidemiology of Indian *S*. Paratyphi A in a global perspective. The burden of Typhoid and Paratyphoid in Indian settings are published separately and can be accessed from the following link  <https://academic.oup.com/jid/issue/224/Supplement_5>  You have raised an important point regarding the lack of functional analysis for the identified mutations. We agree that ‘investigated’ is not an accurate phrase to describe the role of mutation in evolution. Throughout the manuscript we have now adapted the text.  Regarding the agglutination experiment, we have used the monovalent O antiserum that contains factor 1, 2 and 12 (Difco Cat No. 229471). As it the mention of O:2 antisera can be confusing for the reader, we have made corrections throughout the manuscript.  As per the suggestions from other reviewers the section explaining agglutination with O antisera to justify absence of structural change has been removed from the manuscript.  Lack of functional analysis is one of the limitations of this manuscript. This has now been added as limitations of the study but remains in our agenda as future work.  Line No. 304 -309 |
| **Part III**   1. The title should be changed to reflect the conclusions more accurately. The role of genome degradation in emergence and persistence of Paratyphi A lineages is not addressed in this work. | Thank you for pointing out the inappropriate title. We have revised the title to  “Genomic analysis unveils genome degradation events and gene flux in the emergence and persistence of *S*. Paratyphi A lineages” |
| 2. Throughout the authors use non-susceptible where it is common to use ‘resistant’. It is not clear if there is a justification for this distinction. This does make understanding the manuscript more challenging as it is easy to confuse with susceptible, but also seems inappropriate to describe single mutations in QRDR of gyrA as non-susceptible, as these only decrease susceptibility. | Thank you for the suggestion. The phrase ‘non-susceptible’ has been particularly used to address isolates that are not susceptible to quinolones/ciprofloxacin (intermediate/decreased susceptible and resistant). This has been universally used since a large proportion (single mutants) of *Salmonella* isolates fall into intermediate range, as compared to the resistance ranges by CLSI interpretation criteria. To avoid any potential confusion non-susceptible’ was only limited to quinolone susceptibility tests and isolates resistant to first line antibiotics are mentioned as MDR.  Line No. 139 -142 |
| 3. 61-63. The authors predict that mutations in rfb genes would have no impact on vaccination. This is not based on good evidence. Indeed, in lines 211-212 they acknowledge that this needs to be investigated. | Thank you for pointing out the ambiguity of our statement. To avoid misunderstanding, we have reworded our statement in to reflect that this is a hypothesis based on our observation.  Line No. 63 – 64 |
| 4. 65-67. Please clarify how intervention by means of genomic surveillance could avoid *S*. Paratyphi A becoming a threat similar to Typhi? It seems that surveillance could monitor the emergence but not intervene. | Thank you for pointing out that our statement could be understood in this way. As suggested by the reviewer, the word ‘intervention’ has now been changed to ‘monitoring;  Line No. 66 |
| 5. 78-80. Please provide a time frame for this apparent increase. But the data from the India surveillance described in this manuscript would suggest less than 20% and this should be discussed as it disagrees with these previous publications. Was there likely bias in the sampling of this or previous studies that could account for the different conclusions? | Thank you for pointing out these errors and we agree with the reviewer. We have referred the article and rewritten the statement in a proper tone. Line No. 96 - 100  The statement now reads as follows:  “Recent data suggests that the proportion of enteric fever cases caused by *S.* Paratyphi A appear to have significant regional variability in the endemic regions of South Asia. Notably, the contribution of paratyphoid was estimated to be 14.5–37% in population-based studies while hospital-based studies estimate 10.3–54.5% cases of the total enteric fever cases” (Arndt et al., 2014) |
| 6. 111-112. Are these drugs still first line antibiotics considering that treatment primarily targets Typhi that are largely resistant? Some discussion of this in the introduction would help interpret the data here. | Thank you for the assessment. First line agents have now been modified to ‘traditional first-line agents. Details of the first-line agents and the time-line of the development of resistance is available in Introduction.  Line No: 139 – 142 |
| 7. 112. Why 'remained at nearly 98.9%'. Is there data from previous analyses as a comparator? | Thank you, This is revised according to the comment  Line No 139 -142 |
| 8. 136-137. Please clarify how geographical representation affects whether sub-lineage designations are appropriate. Genomic diversity alone seems to be the important criterion for lineage designation. | Thank you, Changes have been made |
| 9. line 151-152. The relevance of reporting ST in this section is not clear. This might be better worked into the section describing Paratypes. | Thank you for your suggestion. We have moved this part to the population structure according to the reviewers comments  Line No. 151 -153 |
| 10. 153-154. Supplementary table 4 states that several isolates were resistant to azithromycin but this is not stated here. | All 441 *S*. Paratyphi A isolates were susceptible to azithromycin and this has now been corrected in Supplementary table 4 (100% susceptibility) |
| 11. 154-157. Please edit to remove repetition from within this section and with the results previously stated earlier. | As per suggestion, we have removed the statements with overlap/redundancy |
| 12. 158-160. These single mutations normally result in a moderate decrease in susceptibility (see PMID: 16895934) which would question the use of the term non-susceptibility. Suggest decreased susceptibility or increased resistance. | As per suggestion, changes have been made throughout |
| 13. 175-176. Suggest highlighting that lineage G was represented by a single strain. This is important as in general these types of observations are only of interest of the polymorphism is present in multiple strains suggesting it is not a transient event. | Changes done  Line No 198 -200 |
| 14. 186-188. What is the evidence that these 'pseudogenes' were associated with the emergence of lineages. Seems to be stated as fact without describing the data. Further, were these identified previously in Zhou et al, 2014 and is there evidence that these are not transient. In general, this section would benefit from more detail. | Thank you for this suggestion. We do not have any evidence on the functional consequence of these frameshift mutations. These observations are based on data generated by analysing *S*. Paratyphi A genome collection representing all major studies and geographical locations (selected spatiotemporally). Lineage defining mutations have been identified by variant calling bioinformatic pipelines and this approach has been widely used in genomics to define lineages. List of all mutations that defined lineages/genotypes are given in Suppl Table No. S5 & S6.  Further evidence on the role of these mutations can be included as a consideration for future study. |
| 15. 193. Why was the lineage G strain excluded? Why are the predicted common ancestor different from that described previously by Zhou et al, 2014? | We also excluded a total of 13 genomes from the dataset including lineage G strain based on our investigations of temporal signal using Tempest. Isolate belong to lineage G (ERR028986) was removed being the outlier and remaining 12 isolates were removed considering the non-availability of collection date.  The predicted year of common ancestor is slightly different due to the advancement of computational software packages (BEAST) that now allow robust and flexible model specification for analysing evolutionary data. The inclusion of best fit models and addition of contemporary isolates after removing the duplicates have improved the overall temporal signal and provided a more accurate prediction |
| 16. 196. Evolutionary rate could be DNA or protein, suggest using nucleotide substitution rate. | We agree with this comment. We have accordingly modified the statement  Line No. 222 |
| 17. 202-204. Only rfbG is present in a large cluster of strains, the other mutations are sporadic and present in multiple isolates that are identical, consistent with transient mutations rather than Darwinian selection. | We agree with the reviewers that rfbG mutations are only present in large cluster of isolates. All isolates except those from lineage D (reference isolate ATCC 9150 belong to same lineage) carried the same mutation. However other mutations cannot be by-passed as sporadic. All these mutations are carried by currently circulating lineages or genotypes as mentioned in the following line. Hence, we could not conclude whether the mutations are transient or positive selection driven.  We have withdrawn the word ‘positive selection’ to avoid ambiguity. Further, to avoid any potential confusion, genotypes carrying such mutations has also been updated in Line No. 229 - 231. |
| 18. 199. Please check that all of these genes are specifically involved in O:2 antigen biosynthesis. I suspect that most are involved in long chain LPS, O2 is a from a branching paratose modification of the O12 backbone. Are any of these genes involved in that modification? | Thank you for pointing out that our statement could be understood in this way. When we mentioned the biosynthesis of O:2 antigen, we meant the whole of *S*. Paratyphi A O antigen synthesis cluster. The agglutination was carried out using monovalent somatic antisera containing factors 1, 2 and 12.  As stated earlier this section has been removed from the manuscript |
| 19. 207-208. Please specify the antigen serum used. From discussion and Methods this is O2. Were there any differences in agglutination with O12, the immune dominant antigen of Paratyphi A? Is serum agglutination sensitive enough to detect changes that could affect immunogenicity? | As mentioned earlier the agglutination was carried out using monovalent somatic antisera containing factors 1, 2 and 12  We agree that the reliance of a qualitative test such as agglutination testing for O antigen expression should be viewed with caution. We also agree that this is a potential limitation of the study. Considering the suggestions from other reviewers, this section has now been removed |
| 20. 214-216. This needs clarification, what does 'evolutionary changes that favor genetic diversity' mean? the intended meaning of 'Evolutionary changes' is not clear but normally means selection of beneficial traits that requires genetic diversity but how this 'favours genetic diversity' is not clear at all. | Thank you for pointing out the ambiguity of our statement. We have reworded the text in the revised manuscript  Line No. 238 |
| 21. 229-230. Please explain why 'The phylogenetic positioning of contemporary S. Paratyphi A isolates in lineage F was unexpected'. | We thank the reviewer for the very interesting comment. Most of the recent studies/publications on the Phylogenetic analysis of *S*. Paratyphi A isolates from South Asia show that the contemporary isolates belong to Lineage A or C (Britto et al, 2018; [10.1371/journal.pntd.0006408](https://doi.org/10.1371/journal.pntd.0006408) & Oo et al., 2019: 10.1093/trstmh/trz053). Reports of isolates from China (10.3201/eid2305.151539) and Cambodia (10.1371/journal.pntd.0005964) also suggested the circulation of isolates belonging to Lineages A & C. Hence it was unusual to find isolates belonging to Lineage F, that were sourced from diverse geographical locations in India. |
| 22. 233-235. Please explain how ‘The emergence of genotype 1.2.2 can be attributed to the acquisition of IncX1 plasmid’. This unequivocal conclusion needs to be supported by some evidence other than coincidence. | Thank you for your suggestion. Pangenome analysis confirmed the presence of IncX1 plasmid similar to pK43 previously sequenced from S. Typhi. (CP029854.1). This plasmid code for a type IV secretion system (T4SS) and present only in isolates belonging to genotype 1.2.2. Since plasmid acquisition is an important mechanism of rapid adaptation and niche expansion in prokaryotes, the acquisition of IncX1 would be the most likely event resulted in the emergence of 1.2.2 previously sporadically reported Lineage F (Genotype 1).  Line No. 202 – 206 |
| 23. 262-264. This section needs a more nuanced discussion since Zhou et al 2014 concluded that there were many transient mutations leading to pseudogenes. Therefore, presumably there are potential examples of lineages and sublineages associated with these that that expanded for reasons other than Darwinian selection for the sequence polymorphism. How did you distinguish between these events? Are the ten pseudogenes listed novel, or were they reported in Zhou et al? | We have not attempted to distinguish whether the lineage/sub-lineage defining mutations (diversifying selection) are advantageous or non- advantageous for *S*. Paratyphi A. As suggested, the lineages or sub-lineages would likely have expanded on reasons other than Darwinian selection but we do not have data from this study to back it.  Regarding pseudogenes, the existing list of 176 and 214 pseudogenes in *S*. Paratyphi A are generated based on two complete genomes ATCC9150 (McClelland et al., 2004) and AKU12601 (Holt et al., 2009). Since the isolate ATCC9150 and AKU12601 is distributed in lineage D and C, respectively, except the three mutations present in isolates belonging to A-E, other seven have not been reported to the best of our knowledge |
| 24. 365-367. How were HDCS and pseudogenes distinguished? There does not appear to have been any functional analysis of HDCSs to confirm that they are pseudogenes. Therefore, ‘HDCS’ would seem more appropriate throughout. | We agree with this and have incorporated your suggestion throughout the manuscript. |
| 25. Discussion: What is known of the potential role of genes affected by potential loss-of-function mutations and how their loss could affect emergence and persistence? | This study deals with genetic changes involved in the evolution of *S*. Paratyphi A that has implications on the emergence and persistence of the bacterium. In this manuscript the role of loss of functional mutation that shape the path of evolution has been systematically investigated and reported. However, such mutations can have varied functional consequences and the effect of mutations in emergence and persistence of bacteria depends on the fitness effects. This is outside the scope our study, but for future studies understanding the contribution of mutations to fitness will be crucial for elucidating the evolutionary paths taken by evolving bacterial populations. |
| **Part I – Summary:**  **Reviewer #3**: This paper describes the genome analysis of a collection of *S*. Paratyphi isolates, predominantly from India. The results describe: the use of a new typing scheme “Paratype” from Bangladesh to define new lineages (as per the Paratype publication); then confirms lineage specific AMR genes as being rare; defines the loss of SPI2 in lineage G (a single isolate); the gain of a PsP3-like phage within sub-groups of lineage 2; the presence of an IncX plasmid in a sub-group of lineage F; some lineage specific mutations with specific acquisition points on the Paratype tree and a statement that the mutations seen in the LPS biosynthetic pathway are unlikely to cause a problem for the vaccine because the organisms still agglutinate the O2 antisera which was used to identify the isolates as Paratyphi A. |  |
| **Part II – Major Issues: Key Experiments Required for Acceptance:**  Reviewer #3: Overall, the paper contains useful data and the analysis provides insights into the strains of *S*. Paratyphi A circulating in the area. However, the analysis does not seem, to this reviewer, to reveal much of interest beyond what is presented in the original Paratype pre-print or is already know about Paratyphi A. The PsP3 phage is interesting, but the analysis is cursory. I cannot see any obvious cargo genes in the sequence nor if all of the 2.3.2/3 lineage isolates have the phage. I tried to look in the Supplementary Table for the genotypes 2.3.2 and 2.3.3 and the phage to see if the lineage remained extant but there are many Paratype genotypes presented as just 2.3 and whilst the AMR genes and the plasmids are there the phage is not.  The treatment of pseudogenes is interesting but needs to be repeated with a more rigorous attention to the impact of each mutation. See <https://academic.oup.com/nar/article/50/9/5158/6576363>  The comment in the abstract "We also identified that the six characteristic missense mutations associated with the lipopolysaccharide (LPS) biosynthesis genes of *S*. Paratyphi A confer only a low structural impact and would therefore have minimal impact on vaccine effectiveness." Is not supported - the argument that the agglutinating antibodies mean there is no structural difference is not a justification as if the antibodies had not agglutinated the bacterial isolate it would have been excluded from the study. | Thank you for the suggestions. We have gone through the entire manuscript carefully and attempted to improve the statements to bring more clarity to the analysis.  The PsP3 phage insert is certainly carried by cargo genes and the presence of integrase gene in the 5’ region has already been annotated. To avoid further confusion, genetic arrangement of the phage inserts with reference (NC_021774) has been reanalysed and Suppl Fig 4 has been improved for better clarity. All isolates of genotype 2.3.2 and 2.3.3 have the PSP3 phage insert and this has been confirmed by Pangenome analysis by Panaroo and Twilight clustering. This has now been added to the Suppl Table S2  We agree that transcriptome and proteome-based techniques mentioned in the manuscript can validate the functional and evolutionary role of pseudogenes. However, in the present study, we have focused only on the genomic epidemiology of *S*. Paratyphi A and the role of genome wide genetic variation in the evolution of currently circulating lineages of the organism.  The structural role of the six characteristic mutations were analyzed using foldX (<https://foldxsuite.crg.eu/>) . The predicted functional effect has been documented in Suppl table S7. We agree that agglutinating with antibodies is not the justification for structural changes. As suggested, we have now removed the statement from the manuscript to avoid any potential confusion. |
| **Part III – Minor Issues: Editorial and Data Presentation Modifications** |  |
| Reviewer #3:  Line 136 et seq: I find this confusing, are the authors saying that the Paratype scheme should be used? | Thank you for pointing out that our statement could be understood in this way. This statement shows the limitations of the current population structure. Hence robust typing scheme such as Paratype should be used. |
| Line 158 "Fluoroquinolone non-susceptibility in dominant lineages (A, C and F) of *S*. Paratyphi A was driven mainly by gyrA-S83F substitutions, with a few isolates harboring gyrA-S83Y (predominantly genotype 160 2.3) variant. Also, a significant number of isolates were fluoroquinolone susceptible with no mutations in the quinolone-resistance-determining region (QRDR), particularly genotype 2.3.1 (Figure 1)." | Thank you for pointing out the ambiguity of our statement. Changes have been made accordingly.  Line No. 181 – 184 |
| The prediction of MIC from QRDR sequence is known to be unreliable, E.g. <https://www.nature.com/articles/s41598-020-64346-0/tables/3> | You have raised an important point here. However, we have not predicted MIC based on the QRDR mutations. |
| Line 178 – “For example, the C4 sub-lineage (genotype 8 179 2.3.2 and 2.3.3) of *S*. Paratyphi A has acquired prophage regions P2/ PSP3 phage that could account for 180 their host specificities (Suppl Fig. 4)” I do not follow this argument - all SPA are host restricted whether they have the phage or not. | We agree with this comment. We have modified the statement accordingly.  Line No. 202 – 203 |
