# Supplementary material for: Genomic analysis unveils genome degradation events and gene flux in the emergence and persistence of S. Paratyphi A lineages
Source: PLoS Pathog. 2023 Apr 28;19(4):e1010650. doi: 10.1371/journal.ppat.1010650 (PMC10171690; doi:10.1371/journal.ppat.1010650)
Supplement: Attachment — Submitted filename: Reviewer_Response.docx [file ppat.1010650.s014.docx]

**Author’s Responses to reviewer questions**

| **Reviewer comments** | **Response** |
| --- | --- |
| **Reviewer #1:** |  |
| **Part I – Summary:**  This is an important study looking at the genomic epidemiology and population structure of a relatively neglected pathogen in India. Genome sequencing and appropriate bioinformatic analyses have been used. I'm very happy with the methodology and interpretation of results. The work is very significant in two main areas. Firstly, in the insights it gives us into evolution of this pathogen and secondly in its relevance to vaccine/diagnostic testing. Overall, the study was well designed and performed. The level of scholarship is what I would expect to see in PLOS Pathogens. | Thank you for your appreciation of our work. Your time and effort in reviewing our manuscript is greatly appreciated. |
| **Part II – Major Issues: Key Experiments Required for Acceptance**  Nothing major but I would like to see confidence intervals (see below) | Thank you for the suggestion. Confidence intervals have now been included. |
| **Part III – Minor Issues: Editorial and Data Presentation Modifications**  Reviewer #1: I'm happy with the author's responses other than their reluctance to provide confidence intervals for their proportions. They do not seem to appreciate that if they repeated their study a hundred times they wouldn't always get the same result for their proportion. A 95% confidence interval will provide a range within which the true proportion will be found in 95 out of 100 repeats of the study. If they have difficulty in doing the calculation an online calculator can be found here:https://sample-size.net/confidence-interval-proportion/  It really isn't difficult to calculate and it is important to acknowledge some degree of uncertainty. | In response to your suggestion, we have added confidence intervals to the proportions given in the Results section.  Page No – 6; Line No. 131 - 136 |
| **Reviewer #2:** |  |
| **Part I – Summary:**  The authors have addressed all relevant comments through edits or removing text that was not supported by the data or analysis. The manuscript now describes additional genome sequences of S. Paratyphi A and an improved higher resolution analysis of the population structure that represents a significant yet limited advance on that published previously. | Thank you, We are very grateful for the time and effort you have dedicated to reviewing our manuscript. |
| **Part II – Major Issues: Key Experiments Required for Acceptance**  No major issues need addressing |  |
| **Part III – Minor Issues: Editorial and Data Presentation Modifications**  No minor issues need addressing |  |
| **Reviewer #3:** |  |
| **Part I – Summary:**  All corrections have been addressed | Thanks for taking the time and effort to review our manuscript. |
| **Part II – Major Issues: Key Experiments Required for Acceptance**  (No Response) |  |
| **Part III – Minor Issues: Editorial and Data Presentation Modifications**  (No Response) |  |
